# Neuralgia.—An Obscure Case

**Published:** 1868-01

**Authors:** N. S. Davis

**Affiliations:** Professor of Practical and Clinical Medicine, in Chicago Medical College


### The Clinique.

### NEURALGIA.—AN OBSCURE CASE.

*Substance of a Clinic in the Medical Wards of Mercy Hospital, December 11th, 1867.*

By N. S. DAVIS, M.D., Professor of Practical and Clinical Medicine, in Chicago Medical College.

The patient before you, gentlemen, is a fair representative of a class of cases which, from their persistence, often tax the resources of the practitioner to the fullest extent. He states that three years since, he was attacked with pain in the left hip, principally in the course of the sciatic nerve, though extending, at times, through to the groin, and a partial loss of motion in the limb. After a few weeks, the pain ceased, and he recovered nearly the perfect use of the limb. During the subsequent two years, he suffered occasional attacks of pain in the same parts, and the muscles of the left leg became weaker than those of the right. During the last year, he has been afflicted with neuralgic pains and morbid sensations almost every day, but extremely variable, both in their location and severity. The pains are more severe in the hips and lumbar portion of the spine than elsewhere; but they frequently change from one to the other, and to the shoulders, arms, legs, especially the heels, the neck, head, and face. In the head, face, and gums, the sensation is described as more of a burning and dryness than acute pain. These morbid sensations, whether of heat, dryness, or acute pain, are extremely changeable, both in their location and severity. They appear to be influenced some by atmospheric conditions, but not in a marked or uniform manner. There is no positive paralysis of either sensation or motion, although the muscles of the left leg are weaker and a little more attenuated than those of the right. His appetite and digestion are good, his bowels regular, and his urine apparently natural in quantity and color. His countenance does not exhibit the physiognomy or expression of severe organic suffering, and his blood and tissues appear to be fairly nourished. I find no marks of disease in his fauces, or on his skin, but the Schneiderian membrane throughout his nostrils is thickened, redder than natural, and his nostrils constantly becoming filled with dry, hard, and black crusts. This condition of the nostrils, he says, has existed since his early boyhood; he being now over 20 years of age. He denies all knowledge of having had any form of syphilitic disease. But of his parents or ancestors I have learned nothing. Such is, briefly, the history of the case before you.

If the suffering of the patient is such as he describes, it is evident that the case must be classed among the neuralgias. To class it thus, however, does not explain its nature, or the essential pathological conditions on which the pains and morbid sensations depend. To aid in arriving at some definite conclusions in reference to this, we may state that all cases of neuralgia may be arranged, pathologically, into three groups:

First. Such as arise from disease or injury directly involving the trunk of one or more nerves.

Second. Such as arise from disease of some part of the central portion of the nervous system.

Third. Such as are caused by morbid conditions of the blood.

In those cases belonging to the first variety, the pain is necessarily limited to the single nerve involved and its branches, as we see in sciatica, tic doloreaux, etc.

In the second class of cases, where the seat of disease is in some part of the cerebral or cerebro-spinal centres, causing neuralgic pains in distant parts, such pains seldom follow the track of any one nerve; but they affect a particular locality or section, such as the forearm, the leg, the foot, the side, etc.

In the third class of cases, the pains are limited to no one nerve or part, but affect many nerves, and usually change with rapidity from one nerve or set of nerves to another, as illustrated in the history of the case now before you.

The morbid conditions of the blood capable of causing neuralgia may be either toxemic or spanemic. That is, poisoned by the presence of some virus imbibed from without, or some effete or excrementitious matter, such as urea, the *materies morbi* of gout, etc.; or such an impoverishment of the blood, in relation to its corpuscles and nutritive constituents, as renders it incapable of affording the elements for healthy nutrition of the nervous structures. Those blood-poisons which, by their immediate or remote effects, are most apt to so modify the sensibility of the nerve structures as to cause persistent and distressing neuralgia, are the syphilitic, the gouty, the uremic, and the koino-malarial.

The effects of these poisons on the properties of the several tissues are not limited to the individuals primarily affected, but may be transmitted, more or less distinctly, to their offspring. This is particularly true in reference to gout and syphilis. Some of the most distressing and obstinate neuralgic affections of the heart, stomach, and extremities accompany the hereditary diatheses transmitted by gouty parents. I know a lady in this city, who was subject to sudden attacks of the most excruciating neuralgic pain in the great toe of one foot. It was accompanied by no swelling or redness, or other traces of inflammation. Her father had suffered many years from gout. It is doubtless true, that a large proportion of the cases of neuralgia arising from the syphilitic poison are caused by a low grade of specific inflammation, either in the neurilemma or in the periosteum lining the bony canals or orifices through which the affected nerves pass; but there are some cases that cannot be attributed to either of these pathological conditions, but, from their changeable character, are evidently dependent on some morbid condition of the blood and nerve sensibility generally. Although the patient before you admits of no known syphilitic influence upon his own person, yet the exceedingly erratic character of his neuralgic pains, the burning dryness of which he complains in his head, face, and neck, in connection with the condition of the Schneiderian membrane of his nostrils, renders it quite probable that his condition is the result of hereditary syphilitic influence. The bridge of the nose is broad and looks a little swollen; and in examining the nostrils, the Schneiderian membrane throughout appears rough, thickened, and constantly secreting a morbid product that dries into hard black crusts.

That a certain degree of syphilitic influence is capable of being propagated to a very remote degree, we have abundant clinical evidence. Hence, we meet with it sometimes under circumstances where it would be least expected. It is not many months since I saw a lady, the mother of a family of children grown to maturity, who was laboring under such symptoms as had led her physician to confidently believe she had serous effusion into the ventricles of the brain. On placing my hand on her head, I discovered the existence of no less than three well-marked pericranial nodes; and all her symptoms of cerebral oppression disappeared under the subsequent use of iodide of potassa and conium.

During the last year, a gentleman, over 70 years of age, was brought here from a neighboring city, where he had led an active business life, and enjoyed a high social position. Several months previously, he had been attacked with what was regarded as apoplexy, or, at least, dangerous congestion of the brain. He was treated actively by men of high standing in the profession, and the first severe symptoms of oppression and stupor were relieved. But he remained with partial paralysis of one arm and leg; severe pains in his head and extremities; impaired memory, frequent mental hallucinations, and almost entire sleeplessness at night. I was told that his case was regarded by his medical attendants as softening of the brain, and mostly beyond the reach of remedial agents. His head being partially bald, I thought one parietal region was more prominent than the other, and, on careful examination, a periosteal thickening, with some tenderness, was found to extend over nearly the whole length of one parietal bone. The patient had noticed this prominence from the commencement of his attack, and it afforded strong evidence that all his cerebral symptoms had been the result of a corresponding disease and tumefaction of the dura mater pressing upon one hemisphere of the brain. All the cerebral and paralytic symptoms disappeared in a few months, under the influence of country air, the steady use of six-grain doses of iodide potassa, aided by a very limited use of alterative doses of bichloride of mercury, and bromide of potassa at night to procure sleep. If this old gentleman had ever had syphilis (which I did not ascertain with certainty), it was, doubtless, more than forty years previously. Such cases, with many others that I might mention, are sufficient to show the necessity of inquiring carefully into the family history of patients laboring under chronic affections of the nervous system; and of observing carefully all local developments that might afford any information concerning the constitutional condition of the patient. In the patient before you, the fact that the morbid condition of the membrane lining the nostrils, already described, has existed from childhood; the peculiarly changeable character of his neuralgic pains and morbid sensations; while the general functions of nutrition, secretion, etc., seem to be well performed, have led me to think that the constitutional vice from which all his distressing symptoms have arisen, is a remote effect of the subtle poison about which we have been speaking.

If this view is correct, we need not expect any permanent advantage from the ordinary remedies for relieving neuralgic pains. His only hope of recovery must be founded on an effort to change his diathesis or constitutional condition. The means best calculated to effect such a change are as follows:

1st. Regular and judicious exercise in the open air, by moderate walking and riding, and, if possible, a change of climate.

2d. A plain, nutritious diet, chiefly of milk, farinaceous articles, and fruits, but from which must be rigidly excluded all fermented and distilled drinks, tobacco, and *strong* tea and coffee.

3d. The use of such alteratives as will be likely to effect a change in the elementary properties of the organized structures of the body, without materially impairing either the plasticity of the blood or the general tone of the tissues.

The first two of these propositions need no comment. The advantages of moderate exercise; a mild and dry climate; plain food, and the exclusion of all nervous stimulants, are obvious to all of you. But what system of medication will effect the third indication? I shall, at present, direct a prescription consisting of,

℞. Iodide Soda, ---------------------------- ʒiij.

Bichlorid. Hydrarg., -------------------- 1 gr.

Fl. Ext. Conium, ------------------------ ℥j.

Simple Syrup,---------------------------

Mint Water,---------------------------āā ℥iss.

Mix, and take a teaspoonful before each meal and at bedtime.

I will have this continued until it produces a change in the secretion from the Schneiderian membrane, or slight traces of the mercurial influence on the gums. When either of these effects are produced, the prescription should be discontinued and the following given in its place:

℞. Iodide Potassa,--------------------------- ʒiij.

Bromide Potassa, ------------------------ ʒvj.

Fl. Ext. Conium, ------------------------ ℥j.

Mint Water, ----------------------------- ℥iij.

Mix, give a teaspoonful from three to four times a-day.

This may be continued for six or eight weeks, unless some unpleasant effects are sooner induced. In the meantime, if the patient becomes in any degree debilitated, as indicated by a feeling of lassitude, diminished appetite, and paleness of the lips, some direct tonic should be given conjointly with the treatment just mentioned. The best tonics I have used in such cases, have been either a teaspoonful of the syrup of pyrophosphate of iron, given half an hour after each meal; or a pill, composed of citrate of iron 2 grs. and strychnia $\frac{1}{30}$ of a grain, given at the same times.

A neglect to insist on good air and a proper use of tonics in conjunction with the usual alteratives, is one cause of failure in the treatment of such cases as the one under consideration.

Besides the hygienic and internal medical treatment which has just been mentioned, I shall direct, with a view of affording temporary relief or mitigating the morbid sensations of the patient, anodyne frictions to the whole length of the spine each night and morning. For this purpose, a liniment composed of the camphorated soap liniment, ℥iv, and veratria, 3 grs., is as effectual as anything I have used. Sometimes, the judicious application of electricity to such cases will produce beneficial results. It should be applied in such a manner, however, as to obtain its tonic effects, rather than irregular shocks. But, gentlemen, the clinic hour has expired, and you will probably have an opportunity to see more of this case in future.

